# P05.49. Development of a model for the conduct of randomized clinical trials of hypnotic intervention

**DOI:** 10.1186/1472-6882-12-S1-P409

**Published:** 2012-06-12

**Authors:** C Kendrick, G Elkins

**Affiliations:** 1 Baylor University, Department of Psychology and Neuroscience, Waco, USA

## Purpose

Research on the efficacy of hypnosis has been limited due to the lack of a sham hypnosis (placebo) for comparison to use as a control in randomized clinical trials. Researchers have used a variety of controls ranging from wait-lists to structured attention, resulting in a lack of blinding of participants and inconsistency. A sham hypnosis methodology would provide a means to compare study results and make aggregate statements regarding hypnosis’ efficacy beyond placebo effects.

The purpose of this study involved two primary aims: 1) to evaluate whether white noise can be considered an “inert” procedure; and 2) to evaluate the feasibility of a model of sham hypnosis that uses white noise as a potential form of “hypnosis” when presented within the hypnotic context.

## Methods

Seventy-five undergraduate students were randomized to one of three groups: hypnosis; sham (white noise presented in the context of hypnosis); or control (white noise in the absence of hypnotic context). Measures of interest involved participants’ ratings of: (1) therapist’s professionalism; (2) the consistency of the environment with hypnosis; (3) subjects’ perception that they received hypnosis; (4) subjects’ evaluation of the procedure as pleasant, relaxing, and beneficial; (5) participants’ perception of the procedure as acceptable, ethical, and effective; and (6) shifts in relaxation resulting from each procedure.

## Results

In each of the variables of interest, subjects who received sham hypnosis and those who received a hypnotic induction demonstrated significant differences from those assigned to the white noise control, with effect sizes ranging from .165 to .852. However, there were no significant differences between participants’ ratings of the sham and hypnosis procedure in any of these domains.

## Conclusion

Results support the feasibility of using white noise as an inert procedure that, given the proper environmental context, can serve as a credible sham hypnosis.

